# Acute Cholecystitis in Very Elderly Patients: Disease Management, Outcomes, and Risk Factors for Complications

**DOI:** 10.1155/2019/9709242

**Published:** 2019-02-03

**Authors:** Alfredo Escartín, Marta González, Elena Cuello, Ana Pinillos, Pablo Muriel, Mireia Merichal, Victor Palacios, Jordi Escoll, Cristina Gas, Jorge-Juan Olsina

**Affiliations:** General Surgery Department, IRBLleida-University Hospital Arnau de Vilanova, Avenue Alcalde Rovira Roure 80, Lleida, Spain

## Abstract

**Background:**

The aim of this study was to evaluate the characteristics, management, and outcomes of acute cholecystitis in patients ≥80 years.

**Methods:**

This was a retrospective analysis of data from a prospective single-center patient registry.

**Results:**

The study population was composed of 348 patients, which were divided into two groups: those younger (Group A) and those older (Group B) than the median age (85.4 years). Although demographic and clinical characteristics of the two groups were similar, the disease management was clearly different, with older patients undergoing cholecystectomy less frequently (*n*=80 46.0% in Group A vs *n*=39 22.4% in Group B; *p* < 0.001). The outcomes in both groups of age were similar, with 30-day mortality of 3.7%, morbidity of 17.2%, and readmissions of 4.2% and two-year AC recurrence in nonoperated patients of 22.5%. No differences were seen between operated and no operated patients. Severe (Grade III) AC was the only independent factor significantly associated with mortality (OR 86.05 (95% CI: 11–679); *p* < 0.001).

**Conclusions:**

In elderly patients with AC, the choice of therapeutic options was not limited by the age per se, but rather by the disease severity (grade III AC) and/or poor physical status (ASA III-IV). In case of grade I-II AC, laparoscopic cholecystectomy can be safely performed and yield good results even in very old patients. Patients with grade III AC present high risk of morbidity and mortality, and the treatment should be individualized. ASA IV patients should avoid cholecystectomy, being antibiotic treatment and cholecystectomy the best option.

## 1. Introduction

Acute cholecystitis (AC) is the most frequent complication of cholelithiasis and one of the most common conditions requiring emergency surgery in the elderly. Cholelithiasis accounts for 90%–95% of all causes of AC, while acalculous cholecystitis accounts for the remaining 5%–10% [1]. Up to 20–40% of asymptomatic patients with gallstones will eventually develop symptoms (annual incidence 1–3%) and in 10–15% of patients with AC will be the first symptomatic manifestation of the disease [2–4].

In Spain, life expectancy currently reaches 80.2 years in men and 86.1 years in women [5]. Given the progressive aging of the population and the increased prevalence of gallstones in older adults, it is understandable why AC is becoming one of the most frequent causes of emergency surgery. The elderly are at high risk to present an episode of AC, and up to 6% of elderly patients will experience severe AC [6]. Laparoscopic cholecystectomy (LC) is currently the gold standard for the management of acute calculous cholecystitis, with preference for early intervention [7, 8]. In the elderly, however, disease characteristics, comorbidities, and poor functional status augment the risks associated with surgical intervention, which may result in increased morbidity and mortality. Most literature consider as elderly patients those whose age is equal or greater than 65 or 75 years [9, 10], though these thresholds may not be the most appropriate from the practical point of view.

We consider that laparoscopic cholecystectomy can be safely performed in many patients of up to 85 years, as previously demonstrated [11–14]. However, the optimal management of AC in patients >85 years is less clear. Some studies suggest that early laparoscopic cholecystectomy yields good results in elderly patients [14–18]; but most of these studies included patients ≥65 years [9, 13, 18–20], with only few studies focused on patients of more advanced age [12, 16, 21]. Even in those few studies, most patients were 80–85 years, with very few patients older than 85, and there was no specific analysis of the population of >85 years.

Under the proposition that when possible cholecystectomy is the best treatment of CA, the objective of the present study was to analyze the characteristics, management, and outcomes of AC in very elderly patients (those aged 80–85 years and those older than 85 years).

## 2. Patients and Methods

### 2.1. Data Source

A prospective analysis of the data collected in a prospective patient registry set up by the General Surgery department of University Hospital Arnau de Vilanova in Lleida, Spain. In 2010, the department modified its protocol for diagnosis, classification, and treatment of AC according to Tokyo guidelines and designed a database for prospective data collection. The data were uploaded into the database using a standard closed-field electronic form; to guarantee patient anonymity, no information that could permit patient identification was registered in the database.

Data from all patients diagnosed with acute calculous cholecystitis who presented at the Emergency department of the hospital Arnau de Vilanova between June 2010 and December 2015 and met selection criteria were included into the database. The criterion for including patients in the database was referral from the emergency room with primary diagnosis of acute calculous cholecystitis. The exclusion criteria were recurrence of AC, AC as secondary diagnosis, acalculous AC, and concomitant acute cholangitis, pancreatitis, gastro-intestinal cancer or bile duct diseases.

For the current study, only the data from patients aged ≥80 years were retrieved from the database and analyzed. History of biliary diseases was defined as prior episodes of acute pancreatitis, acute cholangitis, or obstructive jaundice. Patient's fitness was categorized according to the American Society of Anesthesiologists (ASA) physical status classification score: good physical status (ASA score I-II) and poor physical status (ASA score III-IV). Follow-up of the patients was ended after cholecystectomy in patients who underwent surgery (emergency surgery during an episode of AC or scheduled after the resolution of the symptoms); in nonoperated patients, the minimum follow-up was 24 months. For all patients, complications and mortality of the AC episode occurring within 30 days were taken into account.

The following data were collected: age and gender, disease severity grade, medical history, physical status, history of biliary diseases, presence of choledocholithiasis, microbiological test results, duration of antibiotic therapy, length of inpatient stay, and readmissions. Cholecystectomy was categorized into early (within 48 hours after hospital admission) or delayed (after 48 hours). The complications were evaluated according to Dindo–Clavién scale [13, 22] after having initiated the prescribed treatment.

### 2.2. Diagnosis

Patients who presented in the emergency room with signs and symptoms compatible with AC underwent a blood test (including transaminases, blood cell count, and coagulation study) and abdominal sonography. Abdominal computed tomography scanning or magnetic resonance cholangiography was performed when deemed necessary.

Diagnosis was made based on clinical, laboratory (Murphy's sign, acute upper abdominal pain, right hypochondrial tenderness, fever > 37.5°C and/or white blood cell (WBC) count greater than 10 × 10^9^/L), and ultrasound criteria (thickened > 5 mm and edematous gallbladder, distended gallbladder, positive sonographic Murphy's sign, pericholecystic fluid, and gallstones) [23]. All cases were classified into Grade I (mild), Grade II (moderate), and Grade III (severe) AC according to Tokyo Guidelines [23]: Grade I AC does not meet the criteria for ‘‘grade II” or ‘‘grade III” AC. Grade II AC is associated with any one of the following conditions: (1) elevated WBC count (>18,000 mm^3^), (2) palpable tender mass in the right upper abdominal quadrant, (3) duration of complaints > 72 hours, (4) marked local inflammation (gangrenous cholecystitis, pericholecystic abscess, hepatic abscess, biliary peritonitis, emphysematous cholecystitis). Grade III AC is associated with dysfunction of any one of the following organs/systems: (1) cardiovascular dysfunction: hypotension requiring treatment with dopamine ≥5 mkg/kg per minute, or any dose of norepinephrine, (2) neurological dysfunction: decreased level of consciousness, (3) respiratory dysfunction: PaO_2_/FiO_2_ ratio <300, (4) renal dysfunction: oliguria, creatinine > 2.0 mg/dL, (5) hepatic dysfunction: PT international normalized ratio > 1.5, (6) hematological dysfunction: platelet count <100,000/mm^3^.

### 2.3. Management in the Emergency Room

All the patients were initially assessed in the emergency room, where intravenous fluid therapy, analgesia, proton pump inhibitor, and antimicrobial treatment were started [23]. Later, they were admitted to the hospital ward to start antibiotic treatment or to undergo urgent cholecystectomy, according to the clinical judgement and the patient's wish.

The following antibiotic treatment protocol was followed according Tokyo guidelines and others [23–25]: Grade I: ceftriaxone + metronidazole; Grade II: ceftriaxone + metronidazole (ertapenem in patients > 75 years or with risk factors); Grade III: piperacilin/tazobactam. In case of allergy: gentamycin + metronidazole in Grade I-II; tigecycline + quinolone in Grade III. If no cholecystectomy was performed, the treatment duration was 4–7 days, usually until the normalization of white blood cell count and the reduction of the initial reactive protein C levels by half [26, 27]. If cholecystectomy was performed, the antibiotic treatment was discontinued 24 hours after the surgery, but in case of emphysematosis, vesicular necrosis, perforation or pericholecystic abscess it was maintained for 4–7 days after the surgery [28].

### 2.4. Treatment Options

Laparoscopic cholecystectomy was the standard treatment for patients with grade I or grade II AC, while treatment of grade III AC is defined on an individual basis. However, for various reasons, such as advanced age, significant comorbidity burden, concurrent anticoagulant treatment, concomitant choledocholithiasis, symptom duration at admission >7 days or patient's refusal to be operated on, some patients continue antibiotherapy without undergoing surgery. The on-duty surgeons were experienced surgeons, but for the above reasons or due to lack of experience in hepatobiliary laparoscopic surgery it was sometimes decided to continue medical treatment when there is no life-threatening emergency, given the fact that laparoscopic cholecystectomy in AC may be complex.

Patients with evidence of choledocholithiasis were given antibiotic treatment and underwent urgent endoscopic biliary sphincterotomy to drain the bile duct and to allow for laparoscopic cholecystectomy without the need for bile duct exploration. Ultrasound-guided cholecystostomy was performed in patients who could not be operated on and for whom gallbladder drainage was judged indispensable. The cholecystostomy was clamped when there was a small flow of clean bile, and the catheter was withdrawn in the consultation room after the third week. In patients in which more than 400 cc of bile per day continued to discharge, magnetic resonance cholangiography was performed to rule out obstructive choledocholithiasis.

### 2.5. Surgical Treatment

Laparoscopic cholecystectomy was performed with the patient placed in the French position, using open access (Hasson technique) with umbilical trocar and three accessory ports. In case of conversion or contraindication to laparoscopy, a right subcostal incision was used. The postoperative treatment included antibiotic therapy according to the protocol, analgesia with paracetamol and metamizole, prophylaxis of thromboembolic disease, and proton pump inhibitor administration. Oral intake of water was initiated 6 hours after the intervention, with progressive increase of liquid and food intake according to oral tolerance.

### 2.6. Medicinal Treatment

In admitted patients who did not undergo surgery, the treatment included antibiotic therapy according to the protocol, analgesia with paracetamol and metamizole, prophylaxis of thromboembolic disease, and proton pump inhibitor administration. Oral intake of water was initiated upon admission if the patient did not present nausea or vomiting, with progressive increase of liquid and food intake according to oral tolerance.

### 2.7. Statistical Methods

Since this was an exploratory study, no formal sample size calculations were made. Quantitative variables were expressed as means and standard deviations, and qualitative variable were expressed as frequencies and percentages. Categorical variables were compared using chi square or Fischer's exact test. Continuous variables were compared using Student's *t*-test or Mann-Whitney *U* test (for nonparametric samples). Variables with *p* < 0.2 in the bivariate analyses were included as explanatory variables in multiple logistic regression analysis. Level of statistical significance was set at *p* < 0.05.

## 3. Results

### 3.1. Patient Disposition

A total of 998 patients diagnosed with AC were entered into the local registry between June 2010 and December 2015; 348 (34.5%) of them were ≥80 years, and data from those patients were included in the analysis. Out of those 348 patients, 184 (52.9%) were male; the mean (±SD) age was 85.7 ± 4.0 years, and the median age was 85.4 years (range: 80–99.0). Since 85 years is an age that is frequently taken as the limit to decide actions on the patient, for the sake of analysis, we divided the patients into two equal-size groups: those younger than the median age (Group A: 174 patients aged 80–85.4 years) and those older than the median age (Group B, 174 patients aged 85.4–99.0 years).

### 3.2. Patients' Clinical Characteristics

Clinical characteristics of the two groups are summarized in Table 1. There were no significant differences between the two groups regarding gender, presence of diabetes, physical status, concurrent anticoagulant treatment, history of biliary diseases, or presence of choledocholithiasis. Symptom duration before presenting in the emergency room was similar between the two groups, and there were no differences in the severity of AC (14.4% of patients in Group A and 16.1% in Group B had grade III AC; *p*=0.6). Bile cultures were carried out and found positive with a similar frequency in both groups.

### 3.3. Received Treatment

Although the clinical characteristics, including concurrent anticoagulant treatment, physical status according to ASA and presence of choledocholithiasis, were similar between the two groups, the received treatment was clearly different. Thus, cholecystectomy was performed less often in older patients: out of the total of 119 (34.2%) patients who underwent the procedure, 80 (46.0%) were from the Group A, and only 39 (22.4%) were from the Group B (*p* < 0.001). Laparoscopic approach was used in most (104; 87.4%) cases; the rate of conversion to open surgery was 11.5%, with no significant differences between the groups. Conversely, cholecystostomies were more common among older patients (Table 1): thus, only five (2.9%) patients in Group A underwent this procedure, compared to 20 (11.5%) patients in Group B (*p*=0.002). Although antibiotic treatment was more common among older patients, the duration of the antibiotherapy was similar between the groups (Group A: 7.3 ± 3.4 days; Group B: 7.6 ± 3.3 days; *p*=0.4).

Despite the differences in the disease management, the outcomes were similar in the two cohorts. Thus, there were similar rates of 30-day mortality (Group A: 4 (2.3%) cases; Group B: 9 (5.2%) cases, *p*=0.1), serious complications (Dindo–Calvién grade ≥ II : Group A: 29 (16.7%) patients; Group B: 27 (15.5%) patients; *p*=0.7) and readmissions, as well as the length of hospital stay (Table 1). The rate of AC recurrence was high in both groups (21.7% and 22.3% of nonoperated patients in the groups A and B, respectively; *p*=0.9), and recurrence was even higher if other nontreated complications of cholelithiasis were taken into account (cholecystitis, pancreatitis, or cholangitis) (Group A: 34 (36.2%); Group B: 43 (31.9%); *p*=0.4).

### 3.4. Risk Factors Associated with Mortality and Morbidity

We analyzed which factors were associated with mortality in the overall patient population (Table 2). Out of all the collected clinical and demographic characteristics, the following variables were associated with an increased risk of exitus with *p* < 0.2 in bivariate analyses: presence of diabetes, ASA IV, history of biliary diseases, severe AC (Grade III), cholecystostomy, and absence of antibiotic treatment. However, only severe AC was significantly associated with an increased mortality in multivariate analysis (odds ratio 86.05; 95% CI 11–679; *p* < 0.001).

Similarly, poor physical status (ASA III-IV), ASA IV, history of biliary diseases, severe AC (Grade III), delayed cholecystectomy, any cholecystectomy, cholecystostomy, and absence of antibiotic treatment were associated with an increased risk of serious (Dindo–Clavién ≥ II) complications with *p* < 0.2 in bivariate analyses, but only severe AC, history of biliary diseases, and poor physical status were independent risk factors for complications in multivariate analysis (Table 2).

Severe AC was an important prognostic factor both in case of surgery and nonsurgical treatment. Thus, in case of nonsurgical treatment, mortality was 0 (0%) among patients with mild/moderate AC and 7 (26.9%) among those with severe AC (*p* < 0.001), and complications of Dindo–Clavién grade ≥ II were reported in 18 (8.6%) and 12 (42.6%) (*p* < 0.001). Similarly, in case of surgical treatment, mortality was 1 (1.2%) death among patients with mild/moderate AC and 5 (18.5%) among those with severe AC (*p* < 0.001), and complications of Dindo–Clavién grade ≥ II were reported in 11 (12.8%) and 15 (55.6%) patients (*p*=0.003).

### 3.5. Risk Factors Associated with Severe Grade III AC

Given the importance of severe AC for the prognosis, we analyzed the factors that could potentially contribute to its presence. We found that ASA IV (OR 5.6 (95%CI: 2.7–11.1); *p* < 0.001) and history of biliary diseases (OR 2.7 (95%CI: 1.2–6.0); *p*=0.003) were associated with severe AC in multiple logistic regression analysis.

### 3.6. Treatment Outcomes Stratified according to Age and AC Severity

We analyzed if severe AC was an important risk factor in patients of all ages. Mild/moderate AC (Grade I-II) was equally present in Group A (149 patients; 50.5%) and Group B (146 patients; 49.5%). In group A, morbidity (complications of Dindo–Clavién grade ≥ II) was low both in operated and in nonoperated patients (11 (12.5%) vs 5 (8.2%) cases; *p*=0.4); there were no deaths. Patients in Group B had a higher incidence of serious complications (Dindo–Clavién grade ≥ II) when they were operated (7 (5.8%) nonoperated patients vs 6 (24.0%) operated patients; *p*=0.004), although there was no difference in terms of deaths (0 vs 1 (4.2%) cases, *p*=0.1; Table 3).

Patients with severe AC had worse outcomes both in Groups A and B. In group A, serious complications were common both in operated and nonoperated patients (53% and 50%, respectively, *p*=0.8), and mortality was high (11.8% in operated and 25.0% in nonoperated patients; *p*=0.4). A comparable incidence of deaths and serious complications was observed in Group B. Causes of deaths are shown in Table 4.

## 4. Discussion

Because of the progressive aging of the population, AC in the elderly is becoming an increasingly frequent problem. Although the overall health status of patients of advance age is progressively improving, the presence of comorbidities complicates the choice of treatment. Currently, there is no consensus on the management of AC in very elderly patients, and the related clinical evidence is scarce. In the present study, we analyzed clinical characteristics, treatment, and outcomes of AC in 348 patients aged 80 years or more.

It is known that the comorbidities present in patients over 80 years limit the choice of therapeutic options [12, 13, 16]. Besides, these patients often have a history of previous biliary diseases and concomitant choledocholithiasis at the time of diagnosis [21], which must be taken into consideration when choosing the treatment, since, together with age and patient's physical status, it affects the outcomes [13].

Annually, a large number of patients with AC present in our department, which helps to build a strong expertise in the management of this condition. Whenever possible, we opt for urgent laparoscopic cholecystectomy, since it has been shown to be associated with fewer complications, shorter hospital stay, and cost reduction [12, 22, 29–31]. However, about half of our patients present a significant comorbidity burden [16, 17], about 20% receive concurrent anticoagulation treatment, and 8% have concomitant choledocholithiasis (lower than in other reports) [21], whereas patients of advanced age and their families may oppose surgery, unless there is a vital risk. As a result, only 34% of the patients in our study were operated, with a consequent prolongation of hospital stay [21, 32, 33]. Laparoscopic approach was chosen in 87% of surgeries, which is higher than in previous reports [16, 21], with 11.5% of conversions to open surgery [12, 17]. This conversion rate seems accurate, given the high number of moderate/severe AC in our study, given that inflammatory process makes sometimes very difficult hiliar dissection and AC surgery may be performed by all surgeons with laparoscopic skill, not only hepatobiliary surgeons.

Some authors refer to all patients older than 80 years as “elderly” or “extremely elderly” and consider them high-risk patients [12, 16, 21]. Tokyo guidelines do not consider advanced age as a risk factor per se, although they underline the tendency of older patients to develop severe AC [34]; the flowchart does not make reference to age either, only to surgical risk [23].

Laparoscopic cholecystectomy in older patients was shown to be a safe treatment option even in case of AC [7, 11–13, 15–17], although conservative treatment can also be justified [7, 19]. The latter can initially give good results, but in our study 11 patients out of 240 on conservative treatment had to be urgently operated because of the rapid deterioration. Besides, over the two years of follow-up, 22% of nonoperated patients presented a new episode of AC, and additionally another 11% were readmitted because of other biliary tract problem. These new conditions have been described in literature as generally more severe than the primary conditions [8], and they tend to manifest before a scheduled surgery can be performed.

Some authors propose to adapt watchful waiting in patients with symptomatic cholelithiasis who are older than 90 years and to recur to cholecystectomy only when the condition evolves into AC [17]. Laparoscopic cholecystectomy was shown to be safe in patients older than 80 years [11, 13, 17] and helps to avoid readmissions and complications related to biliary tract problems. The optimal moment for cholecystectomy is not well defined. Although in general an urgent intervention is preferable, the comorbidities and concomitant treatments present in older patients may require prior stabilization and a delay in intervention [9]. We have not found differences in terms of morbidity and mortality between cholecystectomies performed before or after the fifth days since the symptom onset.

In our study, the main risk factor in AC was not the age but the severity of the condition. We found no significant differences in clinical characteristics and outcomes between the two age groups. The results, in terms of morbidity and mortality, were comparable to other published reports on cholecystectomy in octogenarians, even though some of those reports included nonacute conditions or did not include Grade III AC [11–13].

In case of Grade I-II (mild or moderate) AC, the results were good independently of treatment: 41% of patients of 80–85 years were operated, with mortality of 0% and few serious complications. Out of those older than 85 years, only 17% were operated, with the morbidity somewhat higher among those operated and with one death. Given the positive results of cholecystectomy and the high rate of recurrence and related admissions in nonoperated patients, we propose that patients ≥80 years with Grade I-II AC should undergo laparoscopic cholecystectomy, unless contraindicated. When urgent intervention is not possible and no contraindication exists, patients should be scheduled for elective cholecystectomy after discharge.

In case of Grade III (severe) AC, morbidity and mortality rates were high and did not depend on treatment. Although we found no association between age and AC severity, we did find that severe AC was associated with an increased comorbidity burden [17]. Severe AC accounted for most (85%) of our study deaths, which were attributed to the progression of the biliary sepsis present at admission or to preexisting health problems, predominantly cardiac or respiratory [17]. This is why we stress that a rigorous selection of candidates for surgery should be performed to minimize complications. ASA IV patients should avoid cholecystectomy, being antibiotic treatment and cholecystectomy the best option.

In our study carried out in patients ≥80 years, presenting a Grade III AC increased the risk of exitus 86 times, meaning that these patients should be considered high risk from the moment of the diagnosis and should be candidates for admission into an intensive care unit. They require an individualized treatment adjusted to their characteristics in order to reduce morbidity and mortality. We suggest that patients with severe AC and high surgical risk should receive conservative treatment, with cholecystostomy or ultrasound-guided drainage, and should be operated only in case of choleperitoneum or treatment failure.

This study has certain weak points, such as its retrospective character and lack of randomization and control group. Among the strong points of the study, we can name the high number of included patients, structured and homogenous data ensured by the specifically designed database, availability of prospective data (24 months of follow-up), and the use of statistical methods to identify the risk factors.

In conclusion, in elderly patients with AC, the choice of therapeutic options was not limited by the age per se, but rather by the disease severity (Grade III AC) and/or poor physical status (ASA III-IV). In case of Grade I-II AC, laparoscopic cholecystectomy can be safely performed and yield good results even in very old patients; given the high incidence of recurrence of AC and other biliary tract problems, laparoscopic cholecystectomy should be the treatment of choice in the absence of surgery contraindications. If urgent intervention is not possible, we recommend cholecystectomy as soon as possible, usually starting from the 6^th^ week after AC onset. Grade III AC is associated with high morbidity and mortality and requires an individualized treatment: cholecystostomy, laparoscopic cholecystectomy, or conservative treatment.

## Figures and Tables

**Table 1:** Patients' demographic and clinical characteristics, received treatment, and outcomes.

Characteristics	Overall (*n*=348)	80–85.4 years (*n*=174)	85.4–90 years (*n*=174)	*p*
Age, years	85.7 ± 4	82.5 ± 1	89.0 ± 3	<0.001
Male gender	184 (53)	93 (53.5)	91 (52.3)	0.8
Diabetes	104 (30)	52 (29.9)	52 (29.9)	1
ASA III	193 (55.5)	93 (53.4)	100 (57.5)	0.4
ASA IV	43 (12.5)	17 (9.8)	26 (14.9)	0.1
Anticoagulation therapy	77 (22.1)	38 (21.8)	39 (22.4)	0.9
History of biliary disease	40 (11.5)	20 (11.5)	20 (11.5)	1
Choledocholithiasis	28 (8.0)	11 (6.3)	17 (9.8)	0.2
Symptom days at admission	3.1 ± 3.9	2.8 ± 3.3	3.5 ± 4.6	0.1
*AC grade*	—	—	—	0.1
Grade I	106 (30.5)	45 (25.9)	61 (35.1)	
Grade II	189 (54.3)	104 (59.8)	85 (48.9)	
Grade III	53 (15.2)	25 (14.4)	28 (16.1)	
*Received treatment*				
Antibiotics only	210 (60.3)	91 (52.3)	119 (68.4)	0.006
Cholecystostomy (<48 hrs)	25 (7.2)	5 (2.9)	20 (11.5)	0.002
Early cholecystectomy (<48 hrs)	113 (32.5)	74 (42.5)	34 (19.5)	<0.001
*Total cholecystectomy*	119 (34.2)	80 (46.0)	39 (22.4)	<0.001
Laparoscopic cholecystectomy	104 (87.4)	72 (90.0)	32 (82.1)	0.2
Conversion to open surgery	12 (11.5)	9 (12.5)	3 (9.4)	0.6
Days of antibiotic therapy	7.5 ± 3	7.3 ± 3.4	7.6 ± 3.3	0.4
Days of hospital stay	6.5 ± 5	6.0 ± 4.1	6.7 ± 6.0	0.6
30-day complications	60 (17.2)	32 (18.4)	28 (16.1)	0.5
30-day mortality	13 (3.7)	4 (2.3)	9 (5.2)	0.1
30-day readmission	14 (4.2)	10 (5.9)	4 (2.4)	0.1
2-year AC recurrence	49 (22.1)	20 (21.7)	29 (22.3)	0.9
2-year biliary readmission	77 (34.7)	34 (37.0)	43 (33.1)	0.5

**Table 2:** Factors associated with morbidity and mortality.

	Mortality			Complications		
	Survivors (*n*=335)	Deaths (*n*=13)	*p*	Dindo–Clavién < II (*n*=292)	Dindo–Clavién ≥ II (*n*=56)	*p*
Age >85.4 years	165 (49%)	9 (70%)	0.1	147 (50%)	27 (48%)	0.7
Male gender	176 (52%)	8 (61%)	0.5	155 (53%)	29 (52%)	0.8
Diabetes	97 (29%)	7 (54%)	0.05	85 (29%)	19 (34%)	0.4
Poor physical status	145 (43%)	5 (38%)	0.7	150 (51%)	43 (77%)	<0.001
ASA IV	37 (11%)	6 (46%)	<0.001	28 (10%)	15 (27%)	<0.001
History biliary diseases	36 (11%)	4 (31%)	0.02	26 (9%)	14 (25%)	0.001
Choledocholithiasis	27 (8%)	1 (8%)	0.9	23 (8%)	5 (9%)	0.7
AC grade III	41 (12%)	12 (93%)	<0.001	26 (9%)	27 (48%)	<0.001
Cholecystectomy > 48 h	15 (15%)	2 (33%)	0.2	11 (12%)	8 (27%)	0.06
Received treatment						
Antibiotics	210 (63%)	5 (38%)	0.07	192 (66%)	23 (41%)	<0.001
Cholecystostomy	22 (7%)	3 (23%)	0.02	17 (6%)	8 (14%)	0.02
Cholecystectomy	103 (31%)	5 (38%)	0.5	83 (28%)	25 (45%)	0.01

AC grade III	Multiple regression					
	OR (CI 95%)		*p*			
	86 (11–679)		<0.001			

				Multiple regression		
				OR (CI 95%)		*p*
History biliary diseases				3.2 (1.2–8.7)		0.05
Poor physical status				3.2 (1.2–8.7)		0.02
AC grade III				5.1 (2.1–14.9)		0.001

**Table 3:** Characteristics and outcomes of patients with Grade I/II AC.

Group A < 85.4 years: 149 patients (50.5%)	Nonoperated: 88 (58.4%)	Operated: 61 (40.9%)	*p*
Poor physical status	47 (53.4%)	17 (27.9%)	0.002
Cholecystostomy	4 (4.5%)		
Days of hospital stay	5.5 ± 3.1	5.3 ± 3.5	0.7
30-day readmissions	6 (6.8%)	3 (4.9%)	0.6
Two-year AC recurrence	18 (20.7%)	—	
30-day Dindo–Clavién ≥ II	11 (12.5%)	5 (8.2%)	0.4
30-day mortality	0	0	1

Group B > 85.4 years: 146 patients (49.5%)	Nonoperated: 121 (82.8%)	Operated: 25 (17.2%)	*p*

Poor physical status	52 (43.1%)	13 (52.6%)	0.4
Cholecystostomy	11 (9.0%)	—	
Days of hospital stay	5.6 ± 4.6	7.5 ± 7.4	0.09
30-day readmissions	2 (1.7%)	1 (4.2%)	0.4
Two-year AC recurrence	26 (22%)	—	
30-day Dindo–Clavién ≥ II	7 (5.8%)	6 (24.0%)	0.004
30-day mortality	0	1 (4.0%)	0.1
Cause of death		ASA IV-renal failure	

**Table 4:** Characteristics and outcomes of patients with Grade III AC.

Group *A* < 85.4 years: 25 patients (47.1%)	Nonoperated: 8 (32%)	Operated: 17 (68%)	*p*
Poor physical status	4 (50.0%)	8 (47.1%)	0.8
Cholecystostomy	2 (25.0%)	—	
Days of hospital stay	8.1 ± 6.8	10.4 ± 6.5	0.1
30-day readmissions	0	1 (6.7%)	0.9
Two-year AC recurrence	2 (40.0%)	—	
30-day Dindo–Clavién ≥ II	5 (50.0%)	8 (53.3%)	0.8
30-day mortality	2 (25.0%)	2 (11.8%)	0.4
Cause of death	ASA III-septic shock	ASA II-cardiac arrhythmia	
	ASA IV-septic shock	ASA III-septic shock	

Group B > 85.4 years: 28 patients (52.9%)	Nonoperated: 18 (64.3%)	Operated: 10 (35.7%)	*p*

Poor physical status	7 (38.9%)	2 (20.0%)	0.2
Cholecystostomy	13 (72.2%)	—	
Days of hospital stay	12.1 ± 8.4	8.2 ± 7.4	0.2
30-day readmissions	1 (7.7%)	0	0.4
Two-year AC recurrence	3 (25.0%)	—	
30-days Dindo–Clavién ≥ II	8 (44.4%)	6 (60.0%)	0.4
30-days mortality	5 (27.8%)	3 (30.0%)	0.9
Cause of death	ASA III-myocardial infarction	ASA III-myocardial infarction	
	ASA III-respiratory insufficiency	ASA III-septic shock	
	ASA IV-respiratory insufficiency	ASA IV-septic shock	
	ASA IV-septic shock		
	ASA IV-septic shock		

## Data Availability

No data were used to support this study.

## References

[1] Kimura Y., Takada T., Kawarada Y. (2007). Definitions, pathophysiology, and epidemiology of acute cholangitis and cholecystitis: Tokyo Guidelines. *Journal of Hepato-Biliary-Pancreatic Surgery*.

[2] Friedman G. D. (1993). Natural history of asymptomatic and symptomatic gallstones. *American Journal of Surgery*.

[3] Kimura Y., Takada T., Strasberg S. M. (2013). TG13 current terminology, etiology, and epidemiology of acute cholangitis and cholecystitis. *Journal of Hepato-Biliary-Pancreatic Sciences*.

[4] Stinton L. M., Myers R. P., Shaffer E. A. (2010). Epidemiology of gallstones. *Gastroenterology Clinics of North America*.

[5] OECD (2015). Health at a glance 2015: OECD indicators.

[6] Lee S. W., Yang S. S., Chang C. S., Yeh H. J. (2009). Impact of the Tokyo guidelines on the management of patients with acute calculous cholecystitis. *Journal of Gastroenterology and Hepatology*.

[7] Agrawal R., Sood K. C., Agarwal B. (2015). Evaluation of early versus delayed laparoscopic cholecystectomy in acute cholecystitis. *Surgery Research and Practice*.

[8] de Mestral C., Rotstein O. D., Laupacis A., Hoch J. S., Zagorski B., Nathens A. B. (2013). A population-based analysis of the clinical course of 10,304 patients with acute cholecystitis, discharged without cholecystectomy. *Journal of Trauma and Acute Care Surgery*.

[9] Haltmeier T., Benjamin E., Inaba K., Lam L., Demetriades D. (2015). Early versus delayed same-admission laparoscopic cholecystectomy for acute cholecystitis in elderly patients with comorbidities. *Journal of Trauma and Acute Care Surgery*.

[10] Hu Y. R., Pan J. H., Tong X. C., Li K. Q., Chen S. R., Huang Y. (2015). Efficacy and safety of B-mode ultrasound-guided percutaneous transhepatic gallbladder drainage combined with laparoscopic cholecystectomy for acute cholecystitis in elderly and high-risk patients. *BMC Gastroenterol*.

[11] Jesús Ladra M., Paredes J. P., Flores E. (2009). Colecistectomía laparoscópica en pacientes mayores de 80 años. *Cirugía Española*.

[12] Lee S. I., Na B. G., Yoo Y. S., Mun S. P., Choi N. K. (2015). Clinical outcome for laparoscopic cholecystectomy in extremely elderly patients. *Annals of Surgical Treatment and Research*.

[13] Sánchez-Beorlegui J., Soriano P., Monsalve E., Moreno N., Cabezali R., Navarro A. (2009). Colecistectomía laparoscópica en pacientes octogenarios. Estudio comparativo entre dos poblaciones en edad geriátrica. *Cirugía Española*.

[14] Yetkin G., Uludag M., Oba S., Citgez B., Paksoy İ. (2009). Laparoscopic cholecystectomy in elderly patients. *JSLS: Journal of the Society of Laparoendoscopic Surgeons*.

[15] Coenye K. E., Jourdain S., Mendes da Costa P. (2005). Laparoscopic cholecystectomy for acute cholecystitis in the elderly: a retrospective study. *Hepatogastroenterology*.

[16] Dubecz A., Langer M., Stadlhuber R. J. (2011). Cholecystectomy in the very elderly-is 90 the new 70?. *Journal of Gastrointestinal Surgery*.

[17] Loozen C. S., van Ramshorst B., van Santvoort H. C., Boerma D. (2018). Acute cholecystitis in elderly patients: a case for early cholecystectomy. *Journal of Visceral Surgery*.

[18] Riall T. S., Zhang D., Townsend C. M., Kuo Y. F., Goodwin J. S. (2010). Failure to perform cholecystectomy for acute cholecystitis in elderly patients is associated with increased morbidity, mortality, and cost. *Journal of the American College of Surgeons*.

[19] Bueno J., Vaque J., Herrero C. (2007). Acute cholecystitis and laparoscopic cholecystectomy in the elderly. *Cirugía Española*.

[20] McGillicuddy E. A., Schuster K. M., Barre K. (2012). Non-operative management of acute cholecystitis in the elderly. *British Journal of Surgery*.

[21] Nikfarjam M., Yeo D., Perini M. (2013). Outcomes of cholecystectomy for treatment of acute cholecystitis in octogenarians. *ANZ Journal of Surgery*.

[22] Roulin D., Saadi A., Di Mare L., Demartines N., Halkic N. (2016). Early versus delayed cholecystectomy for acute cholecystitis, are the 72 hours still the rule?. *Annals of Surgery*.

[23] Miura F., Takada T., Strasberg S. M. (2013). TG13 flowchart for the management of acute cholangitis and cholecystitis. *Journal of Hepato-Biliary-Pancreatic Sciences*.

[24] Asai K., Watanabe M., Kusachi S. (2011). Bacteriological analysis of bile in acute cholecystitis according to the Tokyo guidelines. *Journal of Hepato-Biliary-Pancreatic Sciences*.

[25] Gomi H., Solomkin J. S., Takada T. (2013). TG13 antimicrobial therapy for acute cholangitis and cholecystitis. *Journal of Hepato-Biliary-Pancreatic Sciences*.

[26] Solomkin J. S., Mazuski J. E., Bradley J. S. (2010). Diagnosis and management of complicated intra-abdominal infection in adults and children: guidelines by the surgical infection society and the infectious diseases society of America. *Clinical Infectious Diseases*.

[27] Guirao X., Arias J., Badía J. M. (2010). Recomendaciones en el tratamiento antibiótico empírico de la infección intraabdominal. *Cirugía Española*.

[28] Loozen C. S., van Santvoor H. C., van Geloven A. A. W. (2017). Perioperative antibiotic prophylaxis in the treatment of acute cholecystitis (PEANUTS II trial): study protocol for a randomized controlled trial. *Trials*.

[29] Reyes M. L., Milanes J. A., Ruiz J. A. (2012). Development of the surgical approach to acute cholecystitis in an emergency surgical unit. *Cirugía Española*.

[30] Wu X. D., Tian X., Liu M. M., Wu L., Zhao S., Zhao L. (2015). Meta-analysis comparing earlyversusdelayed laparoscopic cholecystectomy for acute cholecystitis. *British Journal of Surgery*.

[31] Yamashita Y., Takada T., Strasberg S. M (2013). TG13 surgical management of acute cholecystitis. *Journal of Hepato-Biliary-Pancreatic Sciences*.

[32] Campanile F., Pisano M., Coccolini F., Catena F., Agresta F., Ansaloni L. (2014). Acute cholecystitis: WSES position statement. *World Journal of Emergency Surgery*.

[33] Jang W. S., Lim J. U., Joo K. R., Cha J. M., Shin H. P., Joo S. H. (2014). Outcome of conservative percutaneous cholecystostomy in high-risk patients with acute cholecystitis and risk factors leading to surgery. *Surgical Endoscopy*.

[34] Yokoe M., Takada T., Strasberg S. M. (2013). TG13 diagnostic criteria and severity grading of acute cholecystitis (with videos). *Journal of Hepato-Biliary-Pancreatic Sciences*.

